# Induction of the viable but non-culturable state in bacterial pathogens by household cleaners and inorganic salts

**DOI:** 10.1038/s41598-018-33595-5

**Published:** 2018-10-11

**Authors:** Christian Robben, Susanne Fister, Anna Kristina Witte, Dagmar Schoder, Peter Rossmanith, Patrick Mester

**Affiliations:** 1 Christian Doppler-Laboratory for Monitoring of Microbial Contaminants, Institute for Milk Hygiene, Milk Technology and Food Science, Department of Farm Animal and Public Health in Veterinary Medicine, University of Veterinary Medicine, Veterinärplatz 1, 1210 Vienna, Austria; 2 Institute of Milk Hygiene, Milk Technology and Food Science, Department of Farm Animal and Public Health in Veterinary Medicine, University of Veterinary Medicine, Veterinärplatz 1, 1210 Vienna, Austria

## Abstract

Effective monitoring of microbial pathogens is essential for a successful preventive food safety and hygiene strategy. However, as most monitoring strategies are growth-based, these tests fail to detect pathogenic bacteria that have entered the viable but non-culturable (VBNC) state. The present study reports the induction of the VBNC state in five human pathogens by commercially available household cleaners in combination with inorganic salts. We determined that non-ionic surfactants, a common ingredient in household cleaners, can induce the VBNC state, when combined with salts. A screening study with 630 surfactant/salt combinations indicates a correlation between the hydrophobicity of the surfactant and VBNC induction in *L. monocytogenes*, *E. coli*, *S. enterica* serovar Typhimurium, *S. aureus* and toxin-producing enteropathogenic *E. coli*. Cells that were exposed to combinations of surfactants and salts for 5 min and up to 1 h lost their culturability on standard growth media while retaining their ATP production, fermentation of sugars and membrane integrity, which suggests intact and active metabolism. Screening also revealed major differences between Gram-negative and Gram-positive bacteria; the latter being more susceptible to VBNC induction. Combinations of such detergents and salts are found in many different environments and reflect realistic conditions in industrial and domestic surroundings. VBNC cells present in industrial environments, food-processing plants and even our daily routine represent a serious health risk due to possible resuscitation, unknown spreading, production of toxins and especially their invisibility to routine detection methods, which rely on culturability of cells and fail to detect VBNC pathogens.

## Introduction

Microorganisms are constantly exposed to changing environmental conditions and are forced to employ various survival strategies by reversibly adjusting their physiology^1,2^. This maximizes utilization of resources while structural and genetic integrity are maintained, and increases tolerance to harmful conditions^1,3^. One particular survival strategy in bacteria is the ability to enter a viable but non-culturable (VBNC) state that permits endurance to unfavorable environmental conditions^4,5^. In this regard, the VBNC state is similar to dormancy with the cells retaining an intact membrane, undamaged genetic material and metabolic activity. However, in contrast to dormant cells, VBNC cells are by definition not culturable on routine laboratory media, which are commonly used for hygiene monitoring purposes^6^. Due to their reduced metabolic activity and increased peptidoglycan cross-linking, many VBNC bacteria have higher physical and chemical resistances compared to culturable cells^7–9^. Up until now, approximately 85 species have been shown to enter the VBNC state, including 67 pathogenic bacteria^10^. Understanding resuscitation of these VBNC cells remains challenging to microbiologists. However, in some cases stable resuscitation protocols have been implemented whereby the cells regained their ability to grow and again become virulent^11–13^. So far, the mechanisms for induction and resuscitation of VBNC cells have remained largely unknown. While a few bacteria resuscitate on simply removal of the inducing stress, others are dependent upon specific quorum sensing signals, autoinducers or could only be shown to resuscitate *in vivo*^6,14–16^.

Interestingly, some VBNC pathogens, such as *L. pneumophilia*, enteropathogenic *E. coli*, uropathogenic *E. coli* and *S. typhi*, have maintained their pathogenicity even if they were not able to resuscitate, either due to toxin production or other virulence factors^6,17^.

It has been recently suggested that it is VBNC cells, which are not detected by routine growth-based methods, that account for the fact that only 20% of food-borne diseases can be linked to known pathogens and 80% remain unidentified or unspecified^10^. Indeed, bacterial stress factors present in clinical- or food-processing environments, such as starvation, chlorination, temperature extremes, antibiotic pressure or chemicals such as food preservatives, induce the VBNC state in several human pathogens^18–22^. In most industrial and food-processing environments induction of the VBNC state and complete loss of culturability is a long-term process, which might take up to months to occur under stable conditions^3,10^. In contrast, chlorine treatment, exposure to heavy metals or UV light have been shown to reduce culturability and stimulate VBNC induction rapidly^23,24^.

Recently our group discovered a surprising combinational antimicrobial effect of a non-toxic, non-ionic surfactant and MgCl_2_, which seemed to be highly effective against the foodborne pathogen *L. monocytogenes*^25^. As non-ionic surfactants are now widely used in industrial and household cleaning agents, this novel combinational effect prompted further investigation.

In order to investigate possible VBNC induction and to distinguish VBNC cells from viable and dead cells, we compared the culturability of bacteria with growth-independent viability assays. We combined enzymatic tests for evaluation of metabolic activity and used fluorescence microscopy to analyze cell membrane integrity. Furthermore, we analyzed for culturability and ATP generation in five different pathogens to determine if they retain metabolic activity. Extended testing of metabolic activity was performed for one individual combination of each of the five selected pathogens by use of API 20E test strips that test for carbohydrate fermentation and catabolism of proteins and amino acids, which are essential for viability^26^.

We report VBNC state induction in the five pathogens by exposure to a combination of non-ionic surfactants with salts commonly used in food-processing environments.

As growth-dependent methods are still used overwhelmingly for routine diagnostics, and they fail to detect VBNC pathogens, negative findings may be leading to a false sense of security. The results of this study suggest that adaptation of routine diagnostics to include more complex viability detection techniques is essential in order to correctly evaluate the risk of VBNC pathogens in our daily routine.

## Results

### Non-ionic surfactants induce the VBNC state when combined with inorganic salts

The previously described antimicrobial effect of non-toxic non-ionic surfactants in combination with salts, was further investigated in respect of possible VBNC induction. Initially we investigated possible VBNC induction in *L. monocytogenes* with a few random combinations.

**Figure 1:**
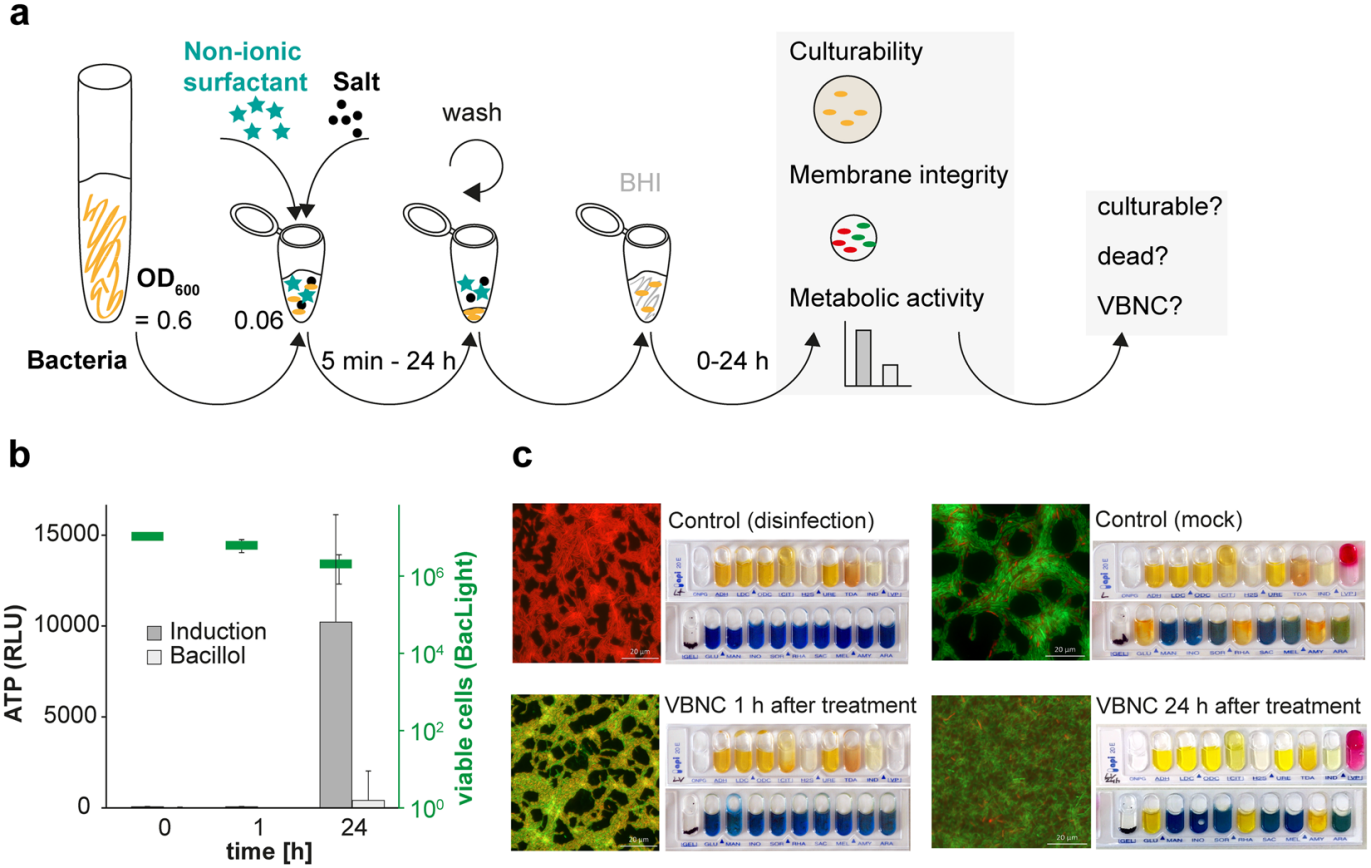
(**a**) Workflow – Induction and confirmation of the viable but non-culturable state, (**b**) ATP determination of non-culturable *L. monocytogenes*. Additionally, the viable cell count (cells/ml) was determined using the LIVE/DEAD^TM^ *Bac*Light^TM^ viability assay (green). (**c**) Development of cells after reuptake in BHI medium; control (mock): culturable cells, negative control (disinfection), VBNC cells 1 h after reuptake in BHI and VBNC cells 24 h after reuptake in BHI; scale: 20 µm, enzymatic activity and ability to ferment sugar by *L. monocytogenes* were measured by the API 20E system.

Therefore, bacteria were centrifuged and exposed to a combination of surfactant with a salt for 1 h. After removing the combinational stress by washing and subsequent resuspension in fresh brain heart infusion broth (BHI) growth medium, tests to determine culturability, membrane integrity and cell metabolism were performed at 0 h, 1 h and 24 h in BHI medium (Fig. 1a).

Figure 1b,c illustrates *L. monocytogenes* after exposure to the non-ionic surfactant Lutensol XP30 in combination with MgCl_2_ for one hour in comparison to cells exposed to the alcohol-based disinfectant Bacillol^®^ AF (disinfection control).

Cells treated with the disinfectant demonstrated red fluorescence in the LIVE/DEAD^TM^ *Bac*Light^TM^ viability assay, indicating damaged cell membranes (Fig. 1c). Further, no *de novo* ATP generation could be observed when incubated for 24 h in BHI medium, confirming cell death (Fig. 1b). In contrast, cells exposed to the combination of Lutensol XP30 and MgCl_2_ retained intact cell membranes, according to the *Bac*Light^TM^ assay. Additionally, if cells were incubated in BHI medium, cell membrane integrity appeared to improve over time, indicated by an increase in green fluorescence intensity without regaining culturability, which also minimizes the risk of regrowth as a few remaining culturable cells would start regrowing within 24 h of incubation in BHI medium (Fig. 1c).

Improvement of VBNC cells was also confirmed by respective fermentation patterns, which show that VBNC cells after 1 h of incubation in BHI medium were not distinguishable from the disinfection control, while after 24 h they showed the same fermentation patterns as culturable *L. monocytogenes* (Fig. 1c). Testing the culturability of cells out of the wells of the API strips revealed that VBNC cells remained non-culturable, while negative and positive controls showed the expected outcome.

It could also be demonstrated that induction of the VBNC state in *L. monocytogenes* occurred rapidly, being observed as early as five minutes after exposure to the Lutensol XP30 / MgCl_2_ combination (Fig. S1).

As for *L. monocytogenes*, we also found surfactant-salt combinations to induce the VBNC state in *E. coli*, *S. enterica*, *S. aureus* and EPEC (Fig. S2). While all pathogens generated ATP after 24 h of incubation in BHI medium, enzymatic activity and fermentation patterns varied.

### Screening of the combinational effect and possible VBNC induction

**Figure 2:**
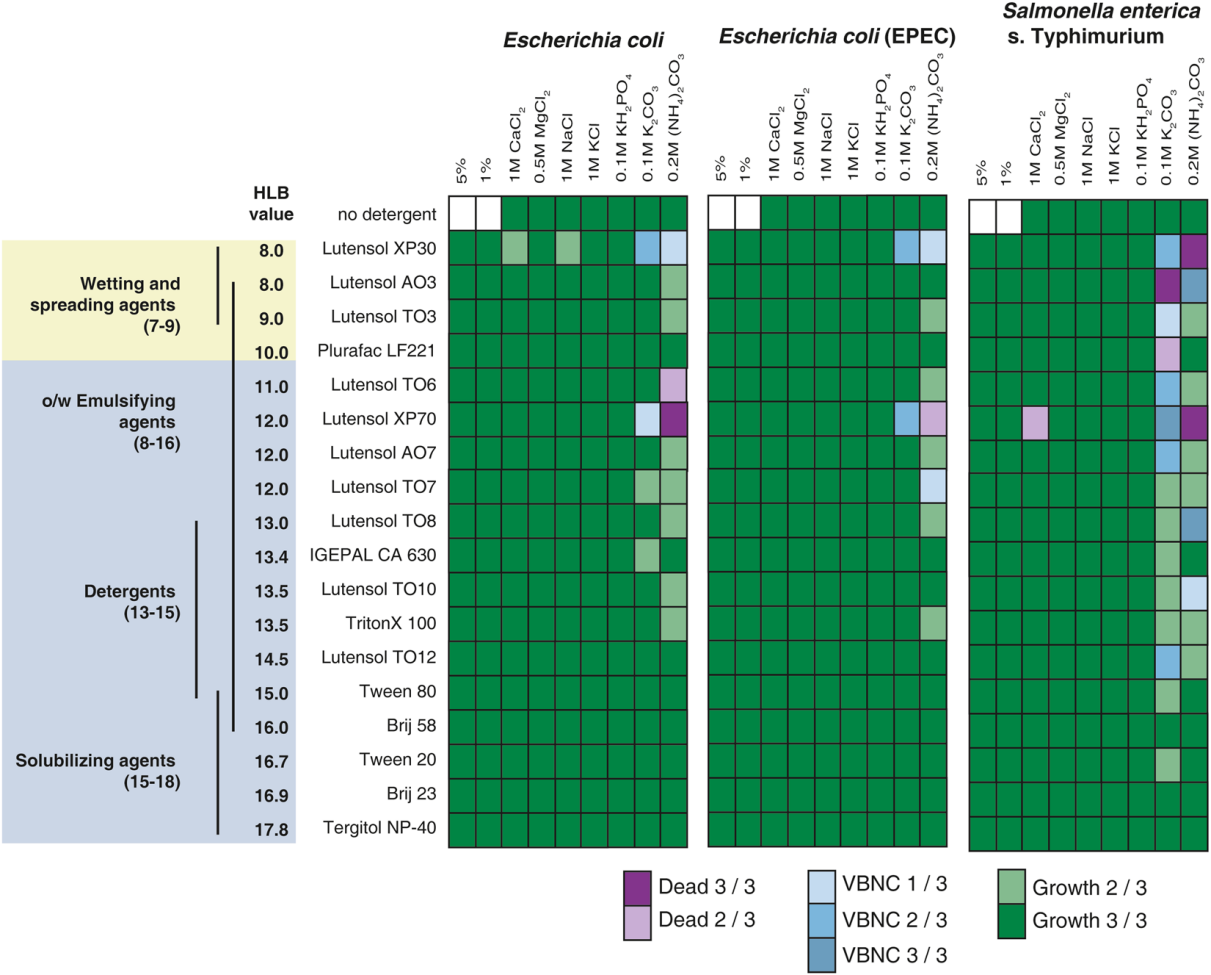
Screening of the combinational effect and induction of the VBNC state in Gram-negative bacteria.

To understand the mechanisms implicated in the combinational effect we performed a screening program with 18 different non-ionic surfactants and seven commonly used salts authorized for use in strictly regulated food-processing environments (Fig. 2). A total of 126 combinations were tested for all five pathogens. The non-ionic surfactants were chosen to cover the hydrophilic-lipophilic balance range (HLB) for non-ionic surfactants, a measure of degrees of hydrophilicity or lipophilicity. The HLB range extends from oil-soluble antifoaming agents at the lower HLB range to water-soluble solubilizing agents at the higher range^27^.

For initial screening of VBNC induction potential, combinations that lead to non-culturability of bacteria after 1 h and 24 h of incubation were further analyzed by measuring ATP generation as an indicator of metabolic activity.

Although no combinatorial effects for non-ionic surfactants with HLB values larger than 15 were found for all tested bacteria, the screening process revealed major differences between Gram-positive bacteria (*L. monocytogenes* and *S. aureus*) and Gram-negative bacteria (*E. coli*, EPEC and *S. enterica*) (Fig. 2).

Eighteen different non-toxic, non-ionic surfactants in combination with seven different salts were tested. Gram-negative cells were classified as dead when the luminescence remained below 100,000 relative light units after 24 h. Above this threshold, cells were regarded as VBNC. This test was performed in triplicate. Combinations were marked as VBNC, if at least one out of three tests led to induction of the VBNC state (while two out of three times, cells remained culturable or dead). For combinations that led to VBNC induction three out of three times, the reproducibility was further tested (Table S1).

For Gram-negative bacteria, culturability was impaired almost exclusively using combinations of surfactants and carbonates, and some of the combinations induced the VBNC state after 1 h incubation. Prolonged exposure of the VBNC bacteria for 24 h increased the number of surfactant/salt combinations that led to cell death, while combinations that did not induce the VBNC state after 1 h did not show this effect.

All combinations leading to VBNC induction three out of three times in initial screening were further analyzed in order to determine reproducibility (Table S1). While stable induction of the VBNC state was confirmed for a number of combinations, results indicate that the induction is a dynamic process that is not completely predictable. Therefore, successful induction of the VBNC state had to be confirmed prior to subsequent analyses.

**Figure 3:**
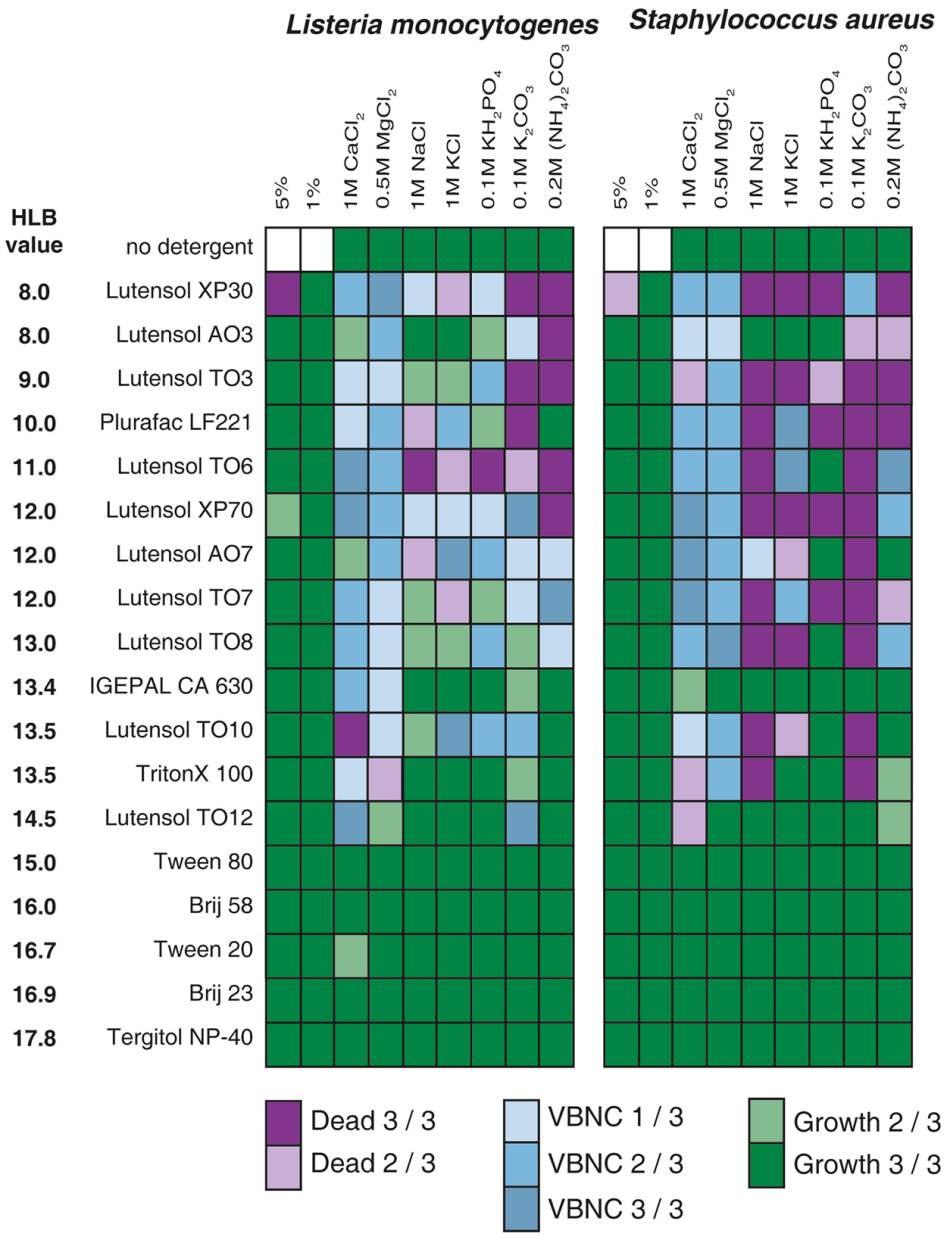
Screening of the combinational effect and induction of the VBNC state in Gram-positive bacteria.

In contrast to results obtained with Gram-negative bacteria, many more combinations affected culturability and induced the VBNC state in Gram-positive bacteria (Fig. 3).

Eighteen different non-toxic, non-ionic surfactants in combination with seven different salts were tested. Gram-positive cells were classified as dead when the luminescence remained below 100,000 relative light units after 24 h. Above this threshold, cells were regarded as VBNC. This test was performed in triplicate. Combinations were marked as VBNC, if at least one out of three tests led to induction of the VBNC state (while two out of three times, cells remained culturable or dead). For combinations that led to VBNC induction three out of three times, the reproducibility was further tested (Table S1).

Although the effect for Gram-negatives was almost exclusively found in combinations with carbonates, there was no salt preference for Gram-positive bacteria. Interestingly, there was almost no combinational effect observable for surfactants with HLB values ≥15.

For the Gram-negative bacteria, prolonged exposure to surfactant/salt combinations for 24 h led more often to cell death. Again, this effect was observed only with bacteria, that entered the VBNC state after 1 h, while culturable bacteria were not affected (Fig. S3). Further, in good agreement with the results for the Gram-negative bacteria, extended analysis of the most stable induction combinations revealed that the induction is a dynamic process that is not completely predictable (Table S1).

### Commercial household cleaners induce the VBNC state in *L. monocytogenes* and *S. enterica*

As non-ionic surfactants are widely used in commercial products, we investigated if common household products could induce the VBNC state. In this respect we tested six different commercial products containing unspecified non-ionic surfactants. After determination of minimum inhibitory concentrations, two products, a dish-washing liquid and a laundry detergent, were further investigated regarding their potential to induce the VBNC state in combination with seven salts, according to our initial screening protocol (Table S2).

While the VBNC state could only be induced in *L. monocytogenes* with the laundry detergent in combination with (NH_4_)_2_CO_3_, the dish-washing liquid induced the VBNC state in both *L. monocytogenes* and *S. enterica* in combination with either KH_2_PO_4_ or MgCl_2_.

Further analysis showed that the VBNC state induced by the dish-washing liquid in *L. monocytogenes* and *S*. *enterica* is comparable to those induced by pure Lutensols as shown previously (Figs S4 and S5). While ATP levels of the dead bacteria remained at the same low level after 24 h, ATP generation by non-culturable *L. monocytogenes* and *S. enterica* increased within 24 h in BHI. They possessed intact cell membranes and the API test confirmed metabolic activity.

Results demonstrate that pathogens can enter the VBNC state after exposure to household-products in combination with inorganic salts.

## Discussion

Induction of the VBNC state by non-toxic, non-ionic surfactants in combination with non-toxic inorganic salt concentrations was confirmed in *L. monocytogenes*, *E. coli*, *S. enterica* serovar Typhimurium, *S. aureus* and toxin-producing enteropathogenic *E. coli*. Moreover, while in the VBNC state, the cell-membrane integrity of the tested bacteria remained intact and their metabolism active.

Screening of 18 non-ionic surfactants combined with seven commonly used salts approved in food-processing industries revealed that the toxic combinational effect and VBNC induction appears to be dependent upon the hydrophilic-lipophilic balance of the surfactants, the respective salt used and the different bacteria. In the case of the surfactants, VBNC induction was only observed with the most hydrophobic surfactants with HLB values between 8 and 14. While the underlying mechanism for the observed effect remains unclear, one possible explanation could be that the membranolytic and cytoplasmic protein destabilizing actions of non-ionic surfactants correspond to their HLB values^28,29^. Relevantly, this might increase membrane permeability, leading to a higher sensitivity of bacterial cells to the combination with salts.

This could also explain why Gram-positive bacteria were much more susceptible to the combinational effect, compared to Gram-negative bacteria, which possess a second cell membrane that serves as an additional barrier. Indeed, Gram-negative bacteria were only susceptible to a few combinations, including carbonates, which are known to increase protein release from cells, inhibit enzyme activities and mediate antibacterial activity via divalent metal binding^30,31^.

However, further investigations are necessary to improve understanding of the combinational effect and the cellular response.

For those combinations that did induce the VBNC state, our results demonstrate that this is a highly dynamic process, which is not completely predictable. Although we could successfully induce VBNC cells after 5 min and 24 h of treatment, the VBNC state has to be confirmed prior to subsequent analyses. Immediately after treatment, VBNC cells show reduced metabolic activity and are barely distinguishable from dead cells. However, we observed a clear and measurable regeneration after reuptake in BHI medium within 24 h, corresponding to increasing metabolic activity and a phenotypic shift of fluorescent cells, indicating improved membrane integrity.

Yet, although there is a clear regeneration process, bacteria could not be resuscitated from the induced VBNC state. While resuscitation of bacterial pathogens has been achieved before, for instance due to simple reversal of the inducing stress, the addition of specific additives or changes to growth media formulations, none of those was successful for the VBNC cells investigated in this study (Table S3)^6,10,32^.

This corroborates results of previous studies which indicate that resuscitation is highly dependent upon induction conditions. Therefore, at this stage it remains unclear as to which measures would allow for resuscitation of cells *in vitro* or if they could resuscitate *in vivo*, as demonstrated for other pathogens^6,10,32^. Even without resuscitation, pathogens in the VBNC state can still be a potential threat to public health due to production of specific virulence factors or toxins^33,34^.

Those non-ionic surfactants that primarily induce the VBNC state are generally used as wetting agents and emulsifiers and are present in many household cleaners, disinfectants, soaps, herbicides, insecticides, biocides and cosmetics, etc. Accordingly, they are found in industrial environments, health care facilities, care communities and domestically. Therefore, we investigated if exposure of bacteria to commercial cleaners with inorganic salts would also induce the VBNC state.

We were able to induce the VBNC state in *L. monocytogenes* and *S. enterica* by replacing the surfactant with a commercially available dish-liquid and a laundry agent. Disturbingly, results demonstrate that pathogens can enter the VBNC state after exposure to these household-products in combination with salts. These highly relevant results reveal how rapidly laboratory research can attain relevance to everyday life.

## Conclusion

Our results demonstrate that the VBNC state in bacteria can be induced almost instantly if they are exposed to a combination of non-ionic surfactants and commonly used inorganic salts, conditions which are widespread throughout industrial, commercial, municipal and domestic environments. We could also show that the VBNC cells induced by such combinations retain their metabolic activity. Although we have not yet been able to resuscitate the bacteria from the induced VBNC state, they still pose a potential public threat. Especially as bacteria in the VBNC state are invisible for growth-based microbiological methods, which as a result may underestimate pathogen risk in routine diagnostics. Further research is necessary to determine the magnitude of this risk and the possible consequences of VBNC pathogens in our daily routine.

## Materials and Methods

### Bacterial Strains and Culture Conditions

*Listeria monocytogenes* EGDe ATCC (1/2a, internal number 2964) was part of the collection of bacterial strains at the Institute of Milk Hygiene, Milk Technology and Food Science, University of Veterinary Medicine, Vienna, Austria. *Escherichia coli* ATCC 25922, *Salmonella enterica* serovar Typhimurium ATCC 14028 and *Staphylococcus aureus* ATCC 6538 were part of the collection of bacterial strains at Merck KGaA, Darmstadt, Germany, and the *eae*-positive enteropathogenic *E. coli* O8:H14 strain (EPEC) was obtained from AGES, Vienna, Austria.

All bacterial strains were grown overnight in brain heart infusion broth (BHI) at 37 °C. Fresh BHI broth was inoculated with 1 ml of the overnight culture and cells were grown to an optical density of 0.6 at 600 nm, making most of the cells be in logarithmic growth phase.

### Induction and confirmation of the VBNC state

In order to investigate possible VBNC induction, 1 ml of early log phase cells (OD_600_: 0.6) were centrifuged for 5 min at 8,000 × g and the pellet resuspended in 1 ml of the respective treatment combination and incubated for 1 h at room temperature (surfactants and salts used are shown in Table 1). Following incubation, samples were centrifuged for 5 min at 8,000 × g and pellets washed once with 1 ml of 1× phosphate buffer solution (PBS). Washed cells were resuspended in 1 ml BHI medium and kept at room temperature for up to 24 h. To investigate possible VBNC induction, culturability, membrane integrity and ATP production were tested directly (0 h), 1 h and 24 h after resuspending cells in fresh BHI growth medium. The 24 h time point also served as a control in order to exclude possible regrowth of remaining low bacterial numbers in the respective samples. To compare VBNC induction with culturable (positive control) and dead cells (negative control), we instead used PBS (positive control) and the disinfectant Bacillol® as negative control for induction. This was performed with *L. monocytogenes, E. coli, S. enterica, S. aureus*, and EPEC. Culturability was tested by plating 5 µl of the culture onto a tryptone soya agar (TSA) plate and incubating overnight at 37 °C.

**Table 1:** Surfactants and salts used for screening the combinational effect; The indicated concentrations are the final concentrations used.

Non-ionic detergents		Company	Salts		Company
Brij 23	1%	Sigma-Aldrich, St. Louis, USA	ammonium carbonate	0.2 M	Merck, Darmstadt, Germany
Brij 58	1%	Sigma-Aldrich, St. Louis, USA	calcium chloride	1 M	Merck, Darmstadt, Germany
IGEPAL CA-630	1%	Sigma-Aldrich, St. Louis, USA	magnesium chloride	0.5 M	Merck, Darmstadt, Germany
Lutensol AO3	1%	BASF, Ludwigshafen, Germany	potassium carbonate	0.1 M	Merck, Darmstadt, Germany
Lutensol AO7	1%	BASF, Ludwigshafen, Germany	potassium chloride	1 M	Fisher Scientific, Hampton, USA
Lutensol TO3	1%	BASF, Ludwigshafen, Germany	potassium dihydrogen phosphate	0.1 M	Merck, Darmstadt, Germany
Lutensol TO6	1%	BASF, Ludwigshafen, Germany	sodium chloride	1 M	Fisher Scientific, Hampton, USA
Lutensol TO7	1%	BASF, Ludwigshafen, Germany			
Lutensol TO8	1%	BASF, Ludwigshafen, Germany			
Lutensol TO10	1%	BASF, Ludwigshafen, Germany			
Lutensol TO12	1%	BASF, Ludwigshafen, Germany			
Lutensol XP30	1%	BASF, Ludwigshafen, Germany			
Lutensol XP70	1%	BASF, Ludwigshafen, Germany			
Plurafac LF 221	1%	BASF, Ludwigshafen, Germany			
Tergitol NP-40	1%	Sigma-Aldrich, St. Louis, USA			
Triton X-100	1%	Merck, Darmstadt, Germany			
Tween 20	1%	Sigma-Aldrich, St. Louis, USA			
Tween 80	1%	Merck, Darmstadt, Germany			

### LIVE/DEAD^TM^*Bac*Light^TM^ bacterial viability fluorescence microscopy

In order to determine if cell membranes of non-culturable bacteria remained intact after exposure to the respective treatment conditions, the LIVE/DEAD^TM^ *Bac*Light^TM^ bacterial viability kit (Molecular Probes, Eugene, USA) was used. Correspondingly, 1 ml of the bacterial sample was centrifuged at 8,000 × g for 5 min, washed with PBS twice and diluted 1:1,000. Subsequently, 1.5 µl of SYTO 9 dye and 1.5 µl of propidium iodide were added to the sample, followed by mixing and 15 min incubation in the dark. After incubation, cells were captured with a 0.2 µm polycarbonate membrane filter (Sterlitec, Kent, USA). The filters were trapped between a slide and a coverslip and used for fluorescence microscopy. This test is based on analysis of cell membrane integrity by fluorescence microscopy. Viable cells with intact membranes are stained green by fluorescent SYTO 9, which can pass the intact or damaged plasma membrane, while propidium iodide can only pass through damaged and disrupted plasma membranes and competes with SYTO 9 for nucleic acid binding sites. Therefore, cells with intact membranes exhibit green fluorescence and cells with damaged membranes exhibit red fluorescence. The latter were considered to be dead. Photographs were taken with the Zeiss Observer Z1 inverted widefield microscope and ZEN 2012 (blue edition) software (Oberkochen, Germany).

### Metabolic activity of non-culturable bacteria

To investigate if cells that had lost their culturability were still metabolically active, ATP was quantified using the BacTiter-Glo^TM^ Microbial Cell Viability Assay (Promega, Madison, USA). Correspondingly, 100 µl of bacterial culture was mixed with 100 µl luciferase reagent and incubated in the dark for 10 min at room temperature. Luminescence was recorded in relative light units (RLU) using a Tecan F100 microplate reader (Männedorf, Switzerland). Sterile BHI medium was used as negative control.

### Enzymatic and metabolic activity of VBNC cells

To investigate the enzymatic activities of VBNC cells and their ability to metabolize sugar, the API 20 E Kit (*BioMérieux*, Marcy l′Étoile, France) was applied. This system can be used to monitor 20 different enzymatic or metabolic activities from a complex sample.

After VBNC induction, cells were resuspended in fresh BHI medium and either used directly or after 24 h of recovery at room temperature. VBNC cells, culturable cells or dead cells were centrifuged at 8,000 × g for 5 min and then resuspended in 0.85% NaCl solution to a density equivalent to McFarland 5 standard. The suspensions were added to the API reaction strips and incubated overnight at 37 °C. After incubation, 5 µl of cell suspension was taken out of the wells showing a color change to verify non-culturability of VBNC cells. Additionally, samples from dead and viable cells were plated randomly as a control. Subsequently, the API colorimetric reagents were added to the appropriate wells and visually evaluated after 10 min of incubation, according to the manufacturers’ instructions.

### Screening of the combinational effect

A screening program with all of the 18 different non-ionic surfactants and seven commonly used inorganic salts was performed three times on three separate days to elucidate the mechanisms underlying the combinational effect (Table 1). For this screening, 100 µl of the respective bacterial strain (*L. monocytogenes, E. coli, S. enterica, S. aureus*, or EPEC, OD_600_: 0.6) was added to 900 µl of the surfactant/salt mixture to reach a final desired concentration (Table 1). For subsequent analysis, samples were taken after 1 and 24 h incubation at room temperature in order to analyze the influence of prolonged exposure. Subsequent analyses were carried out as described previously in “Induction and confirmation of the VBNC state” with the exception that we only used ATP production and plating for viability determination in non-culturable cells at the last tested time point (24 h after reuptake in BHI medium).

Additionally, we tested the stability of the combinational effect, together with VBNC induction, by repeating the procedure on combinations, which led to VBNC induction in the initial screening three out of three times. These combinations were tested again three times on three different days in triplicate (Table S1).

### Development of culturable count during VBNC induction

In order to analyze the induction time of cells exposed to combinations of surfactants and salts, we tracked the development of culturability in *L. monocytogenes* cells during exposure to 1% Lutensol XP30 combined with 0.5 M MgCl_2_. While we used the same procedure as described in “Screening of the combinational effect,” we took samples during induction after 5, 10, 20, 30, 40, 50 and 60 min, washed the samples and tested culturability and VBNC induction as described above.

### Resuscitation of VBNC cells

After induction and confirmation of the VBNC state for the respective bacteria, cells were exposed to different conditions which were shown to facilitate resuscitation previously in different contexts. Therefore VBNC cells, recovering for 24 h in BHI medium, were centrifuged for 5 min at 8,000 × g and resuspended in a distinct condition solution (Table S3). Culturability was tested by plating cells on TSA agar after 24 h and 48 h.

## Electronic supplementary material

Supplementary Information 1
