# Supplementary material for: Induction of the viable but non-culturable state in bacterial pathogens by household cleaners and inorganic salts
Source: Sci Rep. 2018 Oct 11;8:15132. doi: 10.1038/s41598-018-33595-5 (PMC6181970; doi:10.1038/s41598-018-33595-5)
Supplement: Supplementary file 1 — Supplementary Information 1 [file 41598_2018_33595_MOESM1_ESM.pdf]

Supplementary Information for

Induction of the viable but non-culturable state in bacterial pathogens by household cleaners and inorganic salts

Christian Robben[a], Susanne Fister[a], Anna Kristina Witte[a], Dagmar Schoder[a,b], Peter Rossmanith[a,b], Patrick Mester[a*]

[a]Christian Doppler-Laboratory for Monitoring of Microbial Contaminants, Institute for Milk Hygiene, Milk Technology and Food Science, Department of Farm Animal and Public Health in Veterinary Medicine, University of Veterinary Medicine, Veterinärplatz 1, 1210 Vienna, Austria

[b]Institute of Milk Hygiene, Milk Technology and Food Science, Department of Farm Animal and Public Health in Veterinary Medicine, University of Veterinary Medicine, Veterinärplatz 1, 1210 Vienna, Austria

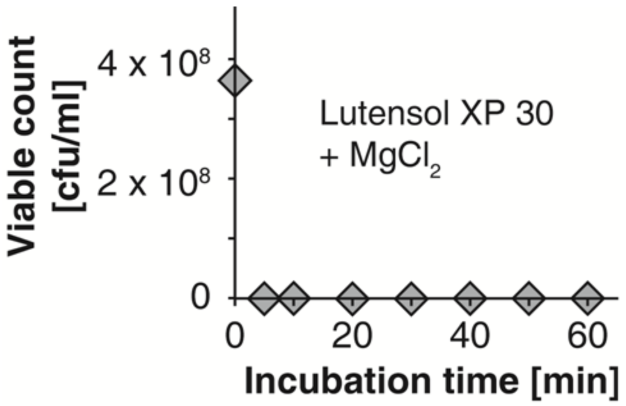

Figure-S1: **Development of the culturable count of *L. monocytogenes***: CFU were determined during exposure to a combination of 1 % Lutensol XP30 and 1 M $MgCl_2$ for up to 60 min of incubation, (detection limit: CFU/ml = 100).

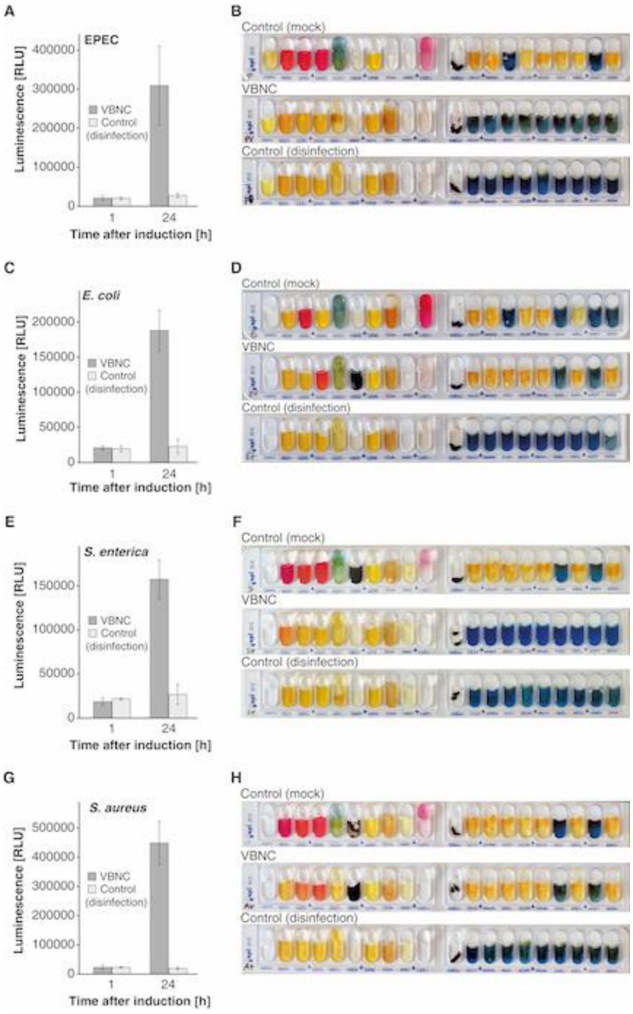

Figure-S2: Confirmation of the VBNC state in different pathogens; A: ATP production of non-culturable enteropathogenic *E. coli* (EPEC); ATP was measured 1 and 24 h after exposure to a

combination of 1 % Lutensol XP30 and 0.1 M K_2CO_3. B: Enzymatic and metabolic activities of EPEC measured by the API 20E system. The same tests were performed with *E. coli* (C/D), *S. enterica* serovar Typhimurium (E/F) and *S. aureus* (G/H).

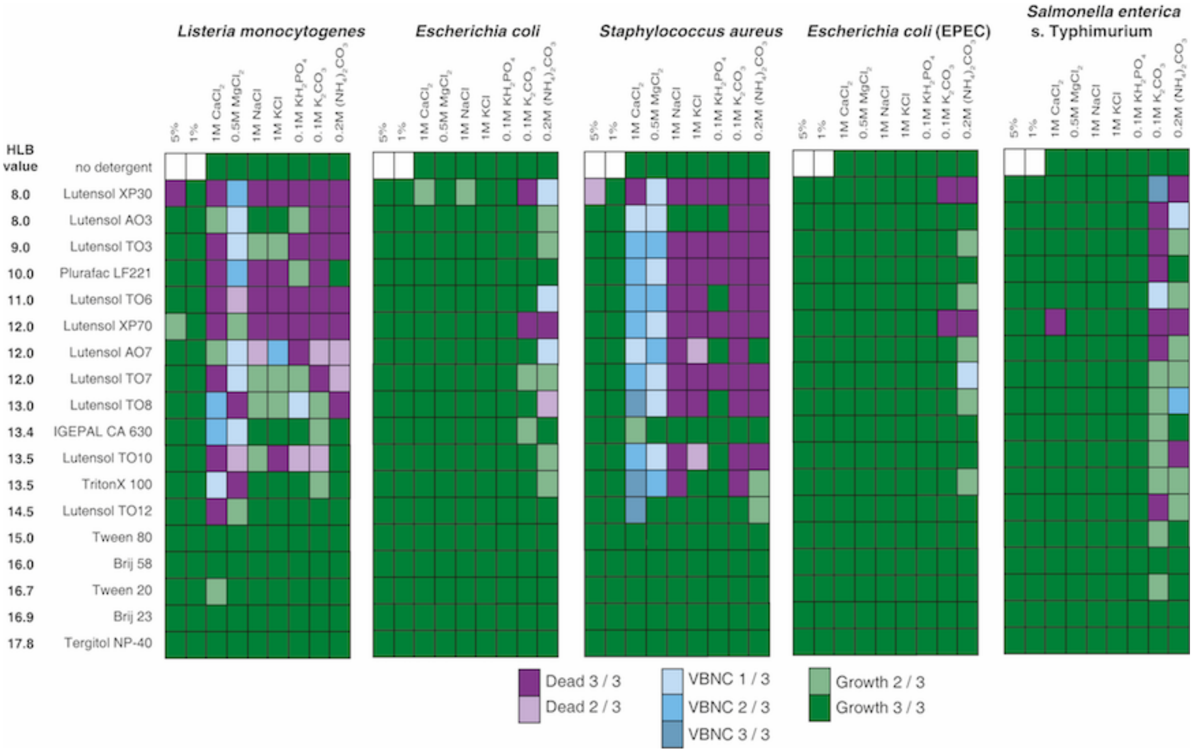

Figure-S3: **Screening of the combinational effect with prolonged exposure to surfactants and salts**

Eighteen different non-toxic, non-ionic surfactants in combination with seven different salts were tested. Surfactants were used at a concentration of 1 % and sorted by HLB value from the lowest at the top to the highest at the bottom. Culturable *S. aureus,* EPEC and *S. enterica* (OD_{600}: 0.6) were exposed to every combination for 24 h at room temperature. Combinations were marked as VBNC, if at least one out of three tests led to induction of the VBNC state (while two out of three times, cells remained culturable or dead). For combinations that led to VBNC induction three out of three times, the reproducibility was further tested (Table S1).

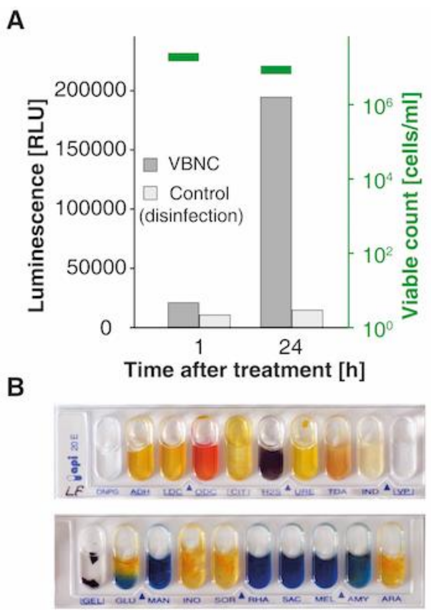

Figure-S4: **Confirmation of the VBNC state in *L. monocytogenes* exposed to dish-washing liquid in combination with KH_2PO_4; A:** ATP determination of non-culturable *L. monocytogenes* after exposure to a combination of 2 % dish-washing liquid and 0.1 M KH_2PO_4 for one hour. Samples were taken 1 h and 24 h after reuptake in BHI medium. Additionally, the viable cell count (cells/ml) was determined using the *Bac*Light^{TM} viability assay. **B**: Enzymatic and metabolic activity of VBNC *L. monocytogenes* induced with dish-washing liquid was measured by the API 20E system. The cell density correlated with McFarland 5 standard.

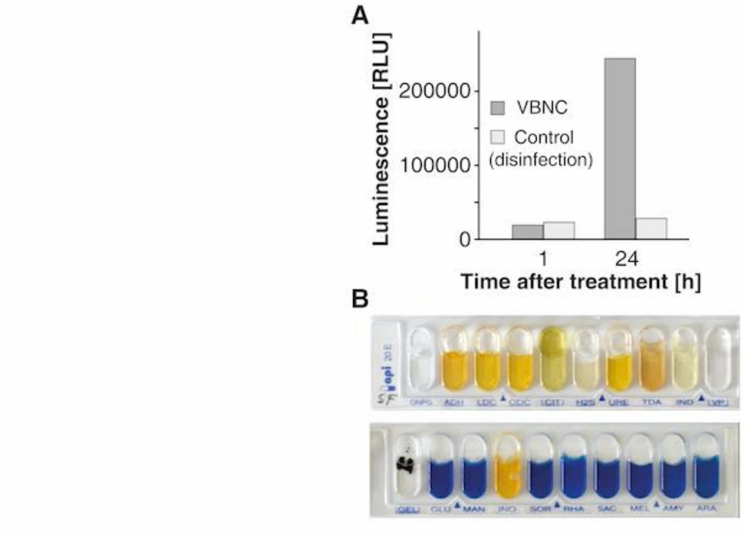

Figure-S5: Induction of the VBNC state by a household cleaner in *S. enterica*; A: ATP generation of non-culturable *S. enterica* after treatment with a dish-washing liquid in combination with $MgCl_2$. Measurements were performed after reuptake in BHI medium and incubation for 1 h and 24 h. B: Enzymatic and metabolic activities of *S. enterica* measured with the API 20E system. The cell density correlated with McFarland 5 standard.

Table-S1: Stability of VBNC induction. Bacterial cell status after exposure to specific combinations of non-ionic surfactants combined with inorganic salts for 1 h at room temperature, which led to VBNC induction three out of three times in earlier experiments were repeated in triplicates (Fig 2 and S3). VBNC 6/9: Out of a total of nine repetitions, the VBNC state was induced six times.

				VBNC	***dead***	***growth***
L. monocytogenes	1 % TO6	+	1 M $CaCl_2$	*6/9*	*3/9*	*0/9*
	1 % XP70	+	1 M $CaCl_2$	*5/9*	*4/9*	*0/9*
	1 % TO12	+	1 M $CaCl_2$	*5/9*	*4/9*	*0/9*
	1 % XP30	+	1 M $MgCl_2$	*9/9*	*0/9*	*0/9*
	1 % AO7	+	1 M KCl	*2/9*	*0/9*	*7/9*
	1 % TO10	+	1 M KCl	*0/9*	*0/9*	*9/9*
	1 % LF220	+	0.1 M K_2CO_3	*0/9*	*0/9*	*9/9*
E. coli	1 % XP30	+	0.1 M K_2CO_3	*9/9*	*0/9*	*0/9*
S. enterica	1 % AO3	+	0.2 M $(NH_4)_2CO_3$	*0/9*	*0/9*	*9/9*
	1 % XP70	+	0.1 M K_2CO_3	*7/9*	*0/9*	*2/9*
	1 % TO8	+	0.2 M $(NH_4)_2CO_3$	*0/9*	*0/9*	*9/9*
S. aureus	1 % XP70	+	1 M $CaCl_2$	*6/9*	*2/9*	*1/9*
	1 % AO7	+	1 M $CaCl_2$	*5/9*	*0/9*	*4/9*
	1 % TO7	+	1 M $CaCl_2$	*4/9*	*0/9*	*5/9*
	1 % TO8	+	1 M $MgCl_2$	*2/9*	*0/9*	*7/9*
	1 % LF221	+	1 M KCl	*0/9*	*0/9*	*9/9*
	1 % TO6	+	1 M KCl	*0/9*	*0/9*	*9/9*
	1 % TO6	+	0.2 M $(NH_4)_2CO_3$	*2/9*	*0/9*	*7/9*

Table-S2: Combinational effect of household cleaners with salt and possible induction of the VBNC state: +: cells remained culturable, -: no growth detected, V: cells entered VBNC state, L: *L. monocytogenes*, E: *E. coli*, S: *S. enterica*, A: *S. aureus*, P: EPEC

	2 % Laundry detergent					2 % Dish liquid				
	L	E	S	A	P	L	E	S	A	P
1 M $CaCl_2$	-	+	+	+	+	-	+	-	-	+
0.5 M $MgCl_2$	-	+	+	+	+	-	+	**V**	+	+
1 M NaCl	-	+	+	+	+	-	+	+	+	+
1 M KCl	-	+	+	+	+	-	+	+	+	+
0.1 M KH_2PO_4	-	+	+	+	+	**V**	+	+	+	+
0.1 M K_2CO_3	-	+	-	+	+	-	+	-	-	+
0.2 M $(NH_4)_2CO_3$	**V**	+	+	+	-	+	+	+	+	+

Table-S3: Resuscitation of VBNC bacteria: VBNC bacteria were exposed to distinct resuscitation conditions for 48 h. - : cells did not resuscitate. Tests were performed in triplicates

**Resuscitation conditions**	***L. monocytogenes***	***E. coli***	***S. enterica***	***S. aureus***	**EPEC**
sterile-filtrated spent medium *L. monocytogenes*	- - -	- - -	- - -	- - -	- - -
sterile-filtrated spent medium *E. coli*	- - -	- - -	- - -	- - -	- - -
sterile-filtrated spent medium *S. enterica*	- - -	- - -	- - -	- - -	- - -
sterile-filtrated spent medium *S. aureus*	- - -	- - -	- - -	- - -	- - -
sterile-filtrated spent medium EPEC	- - -	- - -	- - -	- - -	- - -
autoclaved spent medium *L. monocytogenes*	- - -	- - -	- - -	- - -	- - -
autoclaved spent medium *E. coli*	- - -	- - -	- - -	- - -	- - -
autoclaved spent medium *S. enterica*	- - -	- - -	- - -	- - -	- - -
autoclaved spent medium *S. aureus*	- - -	- - -	- - -	- - -	- - -
autoclaved spent medium EPEC	- - -	- - -	- - -	- - -	- - -
Tween 20 1-10 % in BHI RT	- - -	- - -	- - -	- - -	- - -
Tween 20 1-10 % in BHI 37°C	- - -	- - -	- - -	- - -	- - -
Tween 20 1-10 % in TSB RT	- - -	- - -	- - -	- - -	- - -
Tween 20 1-10 % in TSB 37°C	- - -	- - -	- - -	- - -	- - -
Tween 80 1-10 % in BHI RT	- - -	- - -	- - -	- - -	- - -
Tween 80 1-10 % in BHI 37°C	- - -	- - -	- - -	- - -	- - -

Tween 80 1-10 % in TSB RT	- - -	- - -	- - -	- - -	- - -
Tween 80 1-10 % in TSB 37°C	- - -	- - -	- - -	- - -	- - -
sodium pyruvate 0.3 % in TSB RT	- - -	- - -	- - -	- - -	- - -
sodium pyruvate 0.3 % in TSB 37°C	- - -	- - -	- - -	- - -	- - -
sodium pyruvate 0.3 % in BHI RT	- - -	- - -	- - -	- - -	- - -
sodium pyruvate 0.3 % in BHI 37°C	- - -	- - -	- - -	- - -	- - -
Fraser enrichment medium	- - -	- - -	- - -	- - -	- - -
